# Clinical Impact of Circulating Tumor Cells in Patients with Localized Prostate Cancer

**DOI:** 10.3390/cells8070676

**Published:** 2019-07-03

**Authors:** Lucile Broncy, Patrizia Paterlini-Bréchot

**Affiliations:** 1 INSERM Unit 1151, Faculté de Médecine, Université Paris Descartes, 75014 Paris, France; 2 Laboratoire de Biochimie A, Hôpital Necker-Enfants Malades, 75015 Paris, France

**Keywords:** prostate cancer (PCa), circulating tumor cells (CTC), liquid biopsy

## Abstract

The main issue concerning localized prostate cancers is the lack of a suitable marker which could help patients’ stratification at diagnosis and distinguish those with a benign disease from patients with a more aggressive cancer. Circulating Tumor Cells (CTC) are spread in the blood by invasive tumors and could be the ideal marker in this setting. Therefore, we have compiled data from the literature in order to obtain clues about the clinical impact of CTC in patients with localized prostate cancer. Forty-three publications have been found reporting analyses of CTC in patients with non-metastatic prostate cancer. Of these, we have made a further selection of 11 studies targeting patients with clinical or pathological stages T1 and T2 and reporting the clinical impact of CTC. The results of this search show encouraging data toward the use of CTC in patients with early-stage cancer. However, they also highlight the lack of standardized methods providing a highly sensitive and specific approach for the detection of prostate-derived CTC.

## 1. Introduction

Globally, prostate cancer (PCa) is the most commonly diagnosed type of cancer in men, with 180,890 newly diagnosed cases and 26,120 deaths in the United States (US) in 2016 [1] and estimated 164,690 new cases and 29,430 deaths in 2018 [2]. As the fifth leading cause of cancer death worldwide, PCa accounted globally for 1.6 million newly diagnosed cases and 366,000 deaths in 2015 [1]. Increased risk factors for PCa include genetic predisposition, family history of prostate or breast cancers and older age, with a median age at diagnosis of 72 years [3]. Accordingly, the steady increase in PCa incidence in the US since 1950 appears related to an overall increase in life expectancy. In the US, the lifetime risk of being diagnosed with prostate cancer is of 11% and the lifetime risk of dying of prostate cancer is 2.5% [4]. Importantly, in autopsies of men who died of other causes, more than 20% of men aged 50 to 59 years and over 33% of men aged 70 to 79 years were found to have prostate cancer [5]. At diagnosis, 79% of prostate cancer cases were localized; in 12%, the cancer had spread to regional lymph nodes, and 5% of patients had distant metastasis. The 5-year relative survival rate for localized and regional prostate cancer is 100%, compared with 29.8% for metastatic cases.

PCa is recognized as a genetically heterogeneous disease [6] comprising a large scope of malignancies, from indolent localized cancers that may never progress to rapidly progressing castration-resistant PCa. Currently, the diagnosis of PCa is based on the pathological evaluation of tissue biopsy but the treatment options are determined by risk stratification based on both Gleason score and serum PSA level [7]. For example, high-risk PCa was defined by D’Amico as PSA ≥20 ng/mL and/or biopsy Gleason Score ≥8 and/or clinical stage ≥2c [8].

Elevated serum prostate-specific antigen (PSA) levels are often seen in the context of PCa but can also reflect other prostatic diseases such as benign prostatic hyperplasia, prostatic infection, and prostatic infarction [9]. Relatedly, the low specificity of the PSA screening test has raised concerns in the scientific community regarding the over-diagnosis of PCa [10]. Since the implementation of the PSA screening test into clinical practices in the 1990s, a significant shift towards localized PCa at diagnoses has been observed, with >95% of diagnoses being of clinically localized PCa [11]. Furthermore, only approximately 40 to 50% of patients with elevated PSA testing undergoing a biopsy have prostate cancer. However, a recent study evaluating the ERSPC and PLCO clinical trials has shown that PSA testing reduces the mortality due to PCa by approximately 30% [12]. In cases of localized PCa, radical prostatectomy remains the gold standard treatment option [7]. Importantly, up to 30% of patients treated with radical prostatectomy eventually develop recurrence [13]. More performant risk stratification and prognostic markers are urgently needed to improve the management of patients with localized prostate cancer and identify cases with a high risk of progression.

Circulating tumor cells (CTC) are cells that detach from the primary or secondary tumor sites and invade the bloodstream. As primary actors of the metastatic dissemination, CTC represent a very promising biomarker to aid cancer diagnosis, treatment decision and patient follow-up [14]. In fact, CTC could provide a valuable complement to PSA or other tests with the aim to identify patients with more aggressive cancers. The prognostic value of CTC collected by the epithelial marker-dependent method CellSearch has been established in the context of metastatic PCa [15]. However, the clinical utility of CTC in the context of localized PCa remains unclear. Here, we review studies on non-metastatic prostate cancer to evaluate the potential clinical utility of CTC in localized PCa.

## 2. Materials and Methods

The present review was prepared by selecting English-written research papers describing the detection and/or characterization of circulating tumor cells in the context of localized prostate cancer. To that end, we searched PubMed using the following keywords: “circulating tumor cells” or “circulating cancer cells” and “localized prostate cancer” or “non-metastatic prostate cancer” or “early-stage prostatic carcinoma”. Reviews and studies on liquid biopsy that did not concern localized PCa, as well as studies that did not report on CTC were excluded from the systematic review. Preclinical models, as well as methods for detection of disseminated tumor cells (DTC) in the bone marrow of localized PCa patients, were also excluded. A total of 43 studies were included in the systematic review, as shown in Table 1. It should be noted that several selected studies also reported on locally advanced prostate cancer (T3 and T4) cases. The American Cancer Society defines localized PCa as clinical or pathological tumor stages T1 and T2. PCa with the T1 stage corresponds to a clinically unapparent tumor that is neither palpable nor visible by imaging while T2 corresponds to a tumor that is confined within the prostate and that is either palpable or visible by imaging or demonstrated in radical prostatectomy [16]. In contrast, a pathological tumor stage T3 defines a tumor that has extended through the prostatic capsule and T4 defines a tumor which is invading adjacent organs such as the bladder, sphincter or rectum [16].

## 3. CTC Detection in Non-Metastatic Prostate Cancer

In light of the published data, the extensive variability of CTC detection results in the context of localized PCa appears related to the diversity of distinct methods used for CTC collection/detection. Therefore, the present review will classify the published results depending on the CTC collection/detection methods used.

### 3.1. CTC Detection by RT-PCR in Non-Metastatic Prostate Cancer

Reverse transcription-polymerase chain reaction (RT-PCR) is commonly used to generate amplified cDNA from target mRNA [60]. Therefore, the RT-PCR results largely depend on the specific mRNA that is targeted. As a surrogate test for CTC detection in localized PCa, RT-PCR has mainly been used to target the mRNA of prostate-specific antigen (PSA), prostate-specific membrane antigen (PSMA) and prostate stem cell antigen (PSCA). The only study having compared those three targets as surrogate markers of CTC in localized PCa reported that the detection of PSCA mRNA in blood was the most accurate preoperative predictor of disease-free survival (DFS), probably because PSCA was the only mRNA not detected in 71 non-malignant disorders (PSA detected in 1 and PSMA in 2 of 71 non-malignant disorders) [29]. However, the very low detection rate of PSCA mRNA in that study (detected in only 1 of 43 localized PCa patients) begs caution when interpreting the results. Additionally, Joung et al. reported no association of PSCA mRNA detection with clinical variables on a larger cohort of localized PCa patients [39]. 

Most studies have focused on PSA and PSMA detection in localized PCa and have yielded somewhat contradictory results. Sabile et al. reported that the density gradient separation of mononuclear cells had a higher isolation efficiency than epithelial marker-dependent immunocapture for CTC detection based on PSA RT-PCR [23]. However, by using density gradient separation and PSA RT-PCR, Moreno et al. failed to detect CTC in 4 patients with localized PCa, possibly owing to distinct RT-PCR primer sequences [17]. By studying patients with a mean follow-up of 13.6 months, Olsson et al. determined that PSA mRNA detection was a significant predictor of disease recurrence after prostatectomy [19] while Mejean et al. found a statistical association of PSA-positive RT-PCR with metastasis and recurrence after a follow-up of 26 months [24]. Interestingly, the latter study also tested 11 patients with prostatitis and found positive PSA RT-PCR results in 2 of 11 cases (18%). Other studies including longer follow-up periods reported no correlation of PSA mRNA detection with clinical variables such as overall survival (OS), progression-free survival (PFS), Gleason score, tumor stage or preoperative serum PSA level [20,21,25,27,30,31,34]. Israeli et al. reported that PSMA RT-PCR was more sensitive than PSA RT-PCR in detecting hematogenous tumor cell dissemination but their results were not correlated to clinical variables [18]. Okegawa et al. compared the detection of PSA and PSMA mRNAs as prognostic indicators in a small cohort of 31 localized PCa and determined that PSMA mRNA detection in blood was a significant predictor of PFS after a mean of 16.7 months of follow-up [22]. In contrast, studies including larger casistics and longer follow-up times reported no significant correlation of PSMA mRNA detection with clinical variables [30,34]. Eschwege et al. argued that more specific PSMA RT-PCR primers should be used and that only dual PSA-PSMA-positive blood samples could be considered as reflecting the presence of CTC in blood [36]. Interestingly, the latter study included a rather large cohort of 155 localized PCa patients, more than 100 healthy controls, none of which tested positive for both PSA and PSMA, and 5-year follow-up data showing that the preoperative detection of both PSA and PSMA mRNAs in blood was an independent prognostic factor of disease recurrence [36]. Similarly, Yates et al. showed that both PSA and PSMA mRNA detection improved the prediction of biochemical recurrence over Kattan nomogram [40]. Slawin et al. took a slightly different approach by amplifying the human KLK2 gene, coding for an androgen-regulated protein (hK2) that has an 80% amino acid sequence identity with PSA [26]. Although the Authors determined that RT-PCR-hK2 results allowed for the prediction of lymph node-positive disease, the positivity of their test in 14% of 14 healthy controls indicates a lack of specificity of CTC detection via hK2 RT-PCR [26]. Multiplex RT-PCR approaches may be more efficient in identifying hematogenous prostatic cell dissemination. Yet, the major shortcoming of any RT-PCR approach as a surrogate marker for CTC detection is the related inability to count and further characterize CTC from blood. Furthermore, methodological variability related to different cell extraction methods, primers used for RT-PCR, controls of specificity and sensitivity and the timing of sample collection and storage is expected to account for the heterogeneity of the results obtained and their clinical relevance.

### 3.2. CTC Detection by CellSearch in Non-Metastatic Prostate Cancer

The CellSearch method uses the epithelial cell adhesion molecule (EpCAM) to capture circulating cells and defines CTC as nucleated cells (DAPI+) of epithelial (CK+) and non-hematopoietic (CD45−) origin, which, in fact, better corresponds to a definition of circulating epithelial cells (CEpC). It is important to note that CEpC have been found in the blood of patients with benign colon diseases [61] and benign pancreatic diseases [62]. The lack of specificity of the CellSearch method is exemplified by the finding of CTC in up to 20% of healthy donors tested [33]. 

The preoperative detection of CTC by CellSearch in localized PCa has been reported in 0% to 73% of patients, depending on the study (see Table 1). The fact that distinct cutoff values were used to define CTC positivity in those studies complicates the task to compare their results. For example, Davis et al. and Pal et al. chose to place the cutoff at 1 CTC per 22.5 mL of blood, corresponding to the finding of at least 1 CTC in 3 CellSearch samples of 7.5 mL each [33,47]. In contrast, the majority of studies using CellSearch to detect CTC in localized PCa have used a cutoff of 1 CTC in 7.5 mL [41,42,43,44,49,51]. None of the ten studies using CellSearch to detect CTC in localized PCa have reported a significant correlation of CTC numbers with clinical variables such as OS, PFS, Gleason score, tumor stage or preoperative serum PSA level. 

### 3.3. CTC Detection by Other Marker-Dependent Methods in Non-Metastatic Prostate Cancer

A recent study comparing CellSearch with another EpCAM-dependent method (CellCollector) and the EPISPOT assay (based on the negative enrichment of CTC by leukocyte depletion) reported that only CTC detected by EPISPOT in 58.7% of patients were significantly correlated with clinical parameters such as PSA serum values (*p* < 0.0001) and the clinical tumor stage (*p* = 0.04) [51]. Using the EpCAM-dependent immune-magnetic enrichment of CTC followed by telomerase detection via an enzyme-linked immunosorbent assay, Fizazi et al. detected CTC in 70% of 83 localized PCa patients without false-positive results in 22 healthy controls tested [32]. Unfortunately, the latter study did not include any prognostic evaluation. The EpCAM-dependent microfluidic isolation of CTC has been reported by Stott et al., showing the detection of up to 222 CTC per mL of blood tested and a decline of CTC numbers in 6 of 8 patients 24 h after radical prostatectomy [38]. However, in the latter study, the finding of CTC in healthy controls implied the need for a cutoff value at 14 CTC per mL of blood. By using the AdnaTest, relying on EpCAM and MUC-1 antigens for immune-magnetic isolation and on RT-PCR of Androgen Receptor (AR), c-kit, c-met, ALDH1 and TYMPS for CTC detection, Russo et al. failed to demonstrate a significant association of CTC with clinical parameters [57]. In contrast, Puche-Sanz et al. used cytokeratin-mediated immune-magnetic enrichment of CTC and reported a significant correlation of CTC detection with AR expression in the tumor tissue [54]. The assessment of the AR-V7 splice variant protein in plasma, performed through a capillary nano-immunoassay platform was proposed by Garcia et al. as a surrogate marker for CTC in localized PCa patients [53]. Interestingly, the authors reported a significant correlation of AR-V7 detection with preoperative serum PSA levels and the expression of the stem cell marker CD133. However, the latter study did not perform a longitudinal follow-up of localized PCa patients for further prognostic evaluation [53]. Murray et al. took a different approach by using density gradient isolation and detection of CTC by PSA immunocytochemistry on a large cohort of localized PCa patients [59]. Importantly, the authors reported a significant correlation between CTC detection and clinical variables such as PFS after a long follow-up period of 15 years. However, Murray et al. stored the blood samples at 4 °C during 48 h before analyzing them, which could significantly impact the CTC detection results. Furthermore, the authors neither provided counting of the CTC nor exemplar CTC images.

### 3.4. CTC Detection Following Size-Based Isolation Methods in Non-Metastatic Prostate Cancer

To date, few studies have used size-based separation methods to study CTC from localized PCa patients. Giesing et al. were the first to use blood filtration followed by RT-PCR of PSA and a selection of antioxidant genes (AOX) to detect CTC in 42 localized PCa patients [37]. The authors determined that the detection of antioxidant gene expression in CTC could predict tumor diagnosis with 86% sensitivity and 82% specificity. A few years later, Kolostova et al. used MetaCell® filtration followed by a short-term in vitro culture to identify CTC in 28 of 55 localized PCa patients [45]. Unfortunately, no correlation was found between CTC detection and the clinical parameters. Similarly, Todenhöfer et al. failed to demonstrate a significant correlation with the clinical parameters of CTC detected by fluorescence imaging (EpCAM+ & CD45−) following microfluidic enrichment based on cell-size and deformability [50]. Interestingly, Renier et al. used a similar size-based microfluidic enrichment of CTC followed by the immunofluorescent detection of cytokeratins (CK), PSA and CD45 and reported that some cells did not express epithelial markers (CK) but mesenchymal markers instead (Vim, N-cad), thereby pointing to the process of epithelial to mesenchymal transition (EMT) in circulating prostate cells [56]. Awe et al. also reported on distinct subpopulations of CTC following size-based enrichment and immunostaining for cytokeratins, CD45 and the androgen receptor (AR) but they did not evaluate the clinical impact of those CTC [55]. Efficient risk stratification of localized PCa patients by means of a liquid biopsy was only recently achieved by Miyamoto et al. using the size-based microfluidic enrichment of CTC followed by whole transcriptome amplification and multiplex droplet digital PCR of a panel of 8 genes [58]. By using the differential weighting of 6 genes from the panel, the authors could predict early prostate cancer dissemination in localized disease [58].

## 4. Analysis of the Clinical Value of CTC Detection in Localized Prostate Cancer (Stages T1, T2)

Among the 43 studies targeting non-metastatic patients with PCa included in the present review, 31 investigated the potential clinical impact of CTC detection, looking for a statistical correlation between the detection of CTC and PCa clinical and/or pathological characteristics. Studies reporting on PCa with early (T1–T2) and advanced (T3–T4) stages but without a separate statistical analysis of T1–T2 cancers were further excluded. The details of the remaining 11 studies reporting on the analysis of the clinical value of CTC detection in localized (T1–T2) PCa are shown in Table 2. For clarity, the diagnostic value refers to a test’s ability to identify a disease or a specific condition, with degrees of specificity and sensitivity to express its confidence and accuracy [63]. The predictive value refers to a test’s ability to predict the patient´s response to a specific treatment while its prognostic value identifies risks of progression of the disease independently of a specific treatment [64]. 

The first conclusion of our literature review is that very few studies investigated the clinical impact of CTC selectively in patients with localized prostate cancer. Overall, there is a definite trend toward a value of CTC in correlation with the pathological stage and toward a prognostic and predictive impact of CTC detection in early-stage prostate cancer since several studies have reported significant correlations of CTC numbers with the survival of patients and/or recurrence of the disease after treatment [19,20,24,28,40]. However, those studies used PSA RT-PCR to detect CTC, a method which has yielded contradictory results in other studies [25,27,34], thereby calling for further validation of those results in large cohorts of localized PCa patients. The diagnostic value of CTC detection in early-stage prostate cancer has been less extensively investigated than in metastatic patients. In this regard, the most interesting results come from Giesing et al. with a CTC test demonstrating an 86% sensitivity and an 82% specificity with a 69% positive predictive value and a 92% negative predictive value for PCa diagnosis [37]. However, the latter study reported on both early-stage (T1–T2) and locally advanced (T3–T4) PCa. Puche-Sanz et al. have also investigated the possibility of a diagnostic CTC test for PCa [54]. Yet, with a 14.2% sensitivity and a 78.4% specificity, their test would not further improve on the PSA screening test. The lack of further investigation of a potential diagnostic CTC test could, in fact, stem from the substantial difficulty of detecting CTC in a consistent and specific manner in the context of localized PCa. The presence of circulating prostatic cells in benign prostatic hyperplasia and prostatitis impacts the specificity of certain CTC isolation techniques such as PSA RT-PCR [24,29]. Furthermore, the phenotypic heterogeneity of CTC has been established in the context of metastatic PCa [65]. Additionally, the occurrence of a phenotypic transition (EMT) in CTC from early-stage PCa patients, evidenced by Renier et al. [56], supports the notion of phenotypic heterogeneity among CTC from localized PCa patients as well. The heterogeneity of CTC is relevant to the potential theranostic interest of various CTC tests. Particularly, the expression of the androgen receptor (AR) is of substantial importance for therapy strategy decision in PCa. In fact, a recent review evaluating clinical trials in the context of metastatic PCa determined that the expression of a specific variant of the androgen receptor (AR-V7) was significantly correlated to the limited efficacy of abiraterone and enzalutamide treatments compared with taxane therapy [66]. Whether such an association still holds true in the context of early-stage PCa remains to be demonstrated. Miyamoto et al. also reported a considerable heterogeneity among prostate CTC, including heterogeneous patterns of AR splice variant expression, following microfluidic enrichment and single-cell RNA-seq analyses [67]. So far, only two studies have reported correlations of CTC detection in localized PCa patients with AR expression [54,57]. Puche-Sanz et al. observed a direct association of the expression of AR in the prostatic tissue and the presence of CTC in blood [54]. Russo et al. determined that the expression of AR and TYMS on CTC are frequent events but the implications of such results for a personalized treatment strategy in PCa remain to be elucidated [57]. Further studies are needed to evaluate the potential theranostic utility of CTC in the context of localized PCa.

## 5. Perspectives and Future Directions

The major issue concerning localized prostate cancer is the lack of a suitable marker which could identify benign cases from aggressive prostate cancers. The present study shows a trend toward a possible clinical impact of CTC detection in patients with localized prostate cancer. Despite this trend, the study raises key issues in particular about the technical approaches used, the need for CTC counting and characterization, the sensitivity and specificity controls and the timing of blood sampling. Overall, our analysis encourages the development of a CTC cell-based specific test able to identify and count CTC in a highly sensitive and specific manner in patients with localized cancers and single-cell CTC analyses in patients with localized prostate cancer to specifically study the CTC heterogeneity. It also stimulates the use of a standardized approach to be employed in large clinical studies.

## Figures and Tables

**Table 1:** Studies on circulating tumor cells in patients with non-metastatic prostate cancer.

Study [ref]	N° of Patients (pT Stage)	CTC Method	Cutoff	N° of CTC+ Patients (%)	Median CTC/mL (Range/mL)	Comments	Clinical Impact of CTC
Moreno 1992 [17]	4 (ND ^1^)	PSA RT-PCR	NA ^2^	0 (0%)	NA	17 negative controls (0% positive). Only patients with lymph nodes or metastases tested positive.	ND
Israeli 1995 [18]	13 (7 T2, 5 T3, 1 T4)	PSA & PSMA RT-PCR	NA	PSMA 7 (54%) PSA 0 (0%)	NA	6 years after radical prostatectomy, with undetectable PSA serum levels, patients are PSMA positive	ND
Olsson 1996 [19]	100 (59 T1-T2, 41 T3–T4	PSA RT-PCR	NA	74% (76% T1–T2, 71% T3–T4)	NA	Potential surgical failure defined as a tumor at the surgical margin or extending into the seminal vesicle	Yes
Ennis 1997 [20]	227 (72 T1c, 129 T2, 26 T3)	PSA RT-PCR	NA	61 (26.9%)	NA	Patients treated with prostatectomy had a higher rate of RT-PCR positivity than patients treated with radiation	Yes
Oefelein 1999 [21]	101 (T1–T3a)	PSA RT-PCR	NA	22 (22%)	NA	Median follow-up 22 months	No
Okegawa 1999 [22]	31 (T2–T3)	PSA & PSMA RT-PCR	NA	ND	NA	RT-PCR performed before radical prostatectomy	Yes
Sabile 1999 [23]	10 (ND)	PSA RT-PCR	NA	4 (40%)	NA	Density gradient has higher isolation efficiency than epithelial marker-dependent immunocapture	ND
Mejean 2000 [24]	99 (37 T1, 52 T2, 8 T3, 2 T4)	PSA RT-PCR	NA	33 (33%)	NA	RT-PCR performed preoperatively, 92 controls included (2% scored positive)	Yes
Llanes 2000 [25]	25 (T1–T2b)	PSA RT-PCR	NA	7 (28%)	NA	The best predictors of extraprostatic disease were the biopsy Gleason score and the PSA level.	No
Slawin 2000 [26]	228 (154 T1–T2, 47 T3a, 16 T3b, 11 T4)	hK2 RT-PCR	NA	57 (25%)	NA	14 healthy controls (14% positive). RT-PCR performed before prostatectomy. Association with the risk of metastasis to pelvic lymph nodes (*P* = 0.028).	Yes
Shariat 2002 [27]	224 (T1–T2)	PSA RT-PCR	NA	54 (24%)	NA	RT-PCR performed preoperatively.	No
Bianco 2002 [28]	96 AAM (35 T1, 61 T2) 150 CAM (62 T1, 88 T2)	PSA RT-PCR	NA	26 (27%) AAM 34 (23%) CAM	NA	RT-PCR performed preoperatively.	Yes in AAM
Hara 2002 [29]	44 (26 T1, 15 T2, 2 T3, 1 T4)	PSA, PSMA & PSCA RT-PCR	NA	1 (2.3%) PSA3 (6.8%) PSMA 1 (2.3%) PSCA	NA	RT-PCR performed preoperatively. Positive PSA result in 1 prostatitis case, positive PSMA result in 1 prostatitis and 1 benign prostatic hyperplasia case.	Yes
Thomas 2002 [30]	141 (118 T1c/T2a, 18 T2b/c, 5 T3a)	PSA & PSMA RT-PCR	NA	73 (51.8%)	NA	Only initial PSA and biopsy Gleason score were independent predictors of biochemical failure.	No
Gewanter 2003 [31]	161 (121 T1–T2, 39 T3, 1 Tx)	PSA RT-PCR	NA	22 (20%)	NA	29 months follow-up. Only post-treatment testing predicted for clinical relapse.	No
Fizazi 2007 [32]	83 (38 T1c, 38 T2, 7 T3)	EpCAM + telomerase PCR	NA	58 (70%)	NA	Preoperative CTC detection; 22 healthy controls (0% positive)	ND
Davis 2008 [33]	97 (78 T2, 19 T3)	CellSearch	≥1 CTC /22.5 mL	20 (21%)	0.18 (0.04–2.62)	Preoperative CTC detection; 4 of 20 healthy controls positive for CTC (20%)	ND
Helo 2009 [34]	129 (71 T2, 43 T3a, 13 T3b, 2 T4)	PSA & PSMA RT-PCR	≥80 mRNA/mL	3 (2.6%)	NA	19 healthy controls (0% positive). RT-PCR performed 6 months after surgery for 42 patients and before surgery for 85 patients	No
Maestro 2009 [35]	26 (ND)	CellSearch + CellSpotter Analyzer	≥2 CTC /7.5 mL	4 (15.4%)	ND	106 healthy controls (0% positive)	ND
Eschwe-ge 2009 [36]	155 (T2–T3)	PSA & PSMA RT-PCR	NA	57 (37%)	NA	Preoperative CTC detection; 100 healthy controls (0% positive).	Yes
Giesing 2010 [37]	129 (T1–T4)	Filtration + PSA & AOX RT-PCR	NA	42 (32.5%)	NA	The AOX test was tumour predicting With a positive predictive value of 69% and a negative predictive value of 92%,	Yes
Stott 2010 [38]	19 (T2–T3a)	Microfluidic (EpCAM)	≥14 CTC /mL	8 (42%)	95 (38–222)	6/8 patients with a decline of CTC 24 h after prostatectomy	ND
Joung 2010 [39]	103 (25 T1–T2b, 78 T2c–T3)	PSCA RT-PCR	NA	17 (16.5%)	NA	RT-PCR performed before surgery	Yes
Yates 2012 [40]	92 (61 T1, 31 T2)	PSA & PSMA RT-PCR	NA	63 (68.5%) PSA 68 (78.9%) PSMA	NA	Blood samples taken 1 day preoperatively and 7 days postoperatively	Yes
Lowes 2012 [41]	26 (11 T2, 15 T3)	CellSearch	≥1 CTC /7.5 mL	19 (73%)	ND	Blood drawn before radiation therapy; 7 healthy controls included (0% positive)	Yes
Khurana 2013 [42]	10 (5 T2c, 5 T3a)	CellSearch	≥1 CTC /7.5 mL	1 (10%)	0 (0–0.13)	Blood drawn preoperatively. Very low CTC numbers.	No
Thalgott 2013 [43]	20 (ND)	CellSearch	≥1 CTC /7.5 mL	1 (5%)	0 (0–0.13)	15 healthy controls (0% positive). Shorter overall survival observed only for metastatic patients with ≥ 3 CTC	No
Loh 2014 [44]	36 (9 T1, 14 T2, 13 T3)	CellSearch	≥1 CTC /7.5 mL	5 (14%)	0 (0–0.4)	Blood drawn before therapy. Median follow-up 42 months.	No
Kolostova 2014 [45]	55 (45 T2, 10 T3)	MetaCell® filtration	≥1 CTC /8 mL	28 (52%)	ND	CTC were cultured in vitro for downstream applications for 7–28 days. The captured cancer cells displayed plasticity.	ND
Shao 2014 [46]	40 (26 T2, 13 T3, 1 Tx)	Near-infrared dyes + FACS	≥1 CTC /7.5 mL	39 (97.5%)	10 (0–439)	Blood samples collected preoperatively. Live CTC evidenced by staining with heptamethine carbocyanine dyes	No
Pal 2015 [47]	35 (32 T1–T2, 3 T3)	CellSearch	≥1 CTC /22.5 mL	16 (45%)	0 (0–0.1)	Blood samples drawn before and after surgery. Median follow-up 510 days.	No
Thalgott 2015 [48]	15 (1 T2, 14 T3)	CellSearch	≥1 CTC /20 mL	3 (20%)	0 (0–0.2)	15 healthy controls (0% positive). Median follow-up 44.3 months.	No
Meyer 2016 [49]	152 (95 T2, 40 T3a, 17 T3b)	CellSearch	≥1 CTC /7.5 mL	17 (11%)	0.13 (0.13–13.3)	Blood samples collected preoperatively. Median follow-up 48 months.	No
Toden-höfer 2016 [50]	50 (37 T2, 13 T3)	Microfluidic (size, deforma-bility)	≥1 CTC /2 mL	25 (50%)	4.5 (0.5–208.5)	Pancytokeratin positive CTC showed expression of androgen receptor.	No
Kuske 2016 [51]	86 (37 T1, 45 T2, 4 T3)	CellSearch EPISPOT CellCollector	≥1 CTC /7.5 mL	37% CS 54.9% CC 58.7% EPI	0.24 (0.13–1.3) CS 0.32 (0.13–1.6) CC 0.4 (0.13–1.7) EPI	Blood drawn preoperatively. CTC detected by EPISPOT correlated to tumor stage, no correlation found with CellSearch (CS) or CellCollector (CC)	Yes
Tsumura 2017 [52]	59 (26 T1c–T2a, 15 T2b-c, 17 T3, 1 T4)	CellSearch	≥1 CTC /7.5 mL	0 (0%) preoperative 7 (11.8%) intraoperative	ND	Blood drawn both before and during surgery, with detection of CTC only during surgery.	No
Garcia 2017 [53]	16 (ND)	AR-V7 protein in serum samples	AR-V7 protein detection	3 (18.7%)	NA	CD133 expression in CTC was higher among AR-V7 positive cases vs. AR-V7 negative	ND
Puche-Sanz 2017 [54]	86 (T1–T2)	CK immune-magnetic	≥1 CTC /10 mL	16 (18.6%)	0 (0–0.4)	Blood samples collected before biopsy. Analysis of AR expression in tumor tissue.	Yes
Awe 2017 [55]	41 (T1–T4)	Filtration + immunostaining CK, CD45, AR	≥1 CTC /3 mL	41 (100%)	ND	Blood samples collected before prostatectomy	ND
Renier 2017 [56]	1 (ND)	Microfluidic vortex chip (size-based) + immuno-staining CK, CD45, PSA	>3.37 CTC/7.5 mL = >0.45 CTC/mL	1 (100%)	1.5	Some double positive cells (CK+, CD45+) found but counted as WBC. Some cells did not express epithelial markers (CK, PSA) but mesenchymal instead (Vim, N-cad)	ND
Russo 2018 [57]	47 (31 T2, 16 T3a)	AdnaTest Prostate-Cancer	0.15 ng /µL for AR, c-kit, c-met, ALDH1, TYMS. 0.25 ng /µL for Akt-2 & PI3Kα	12 (25.5%)	NA	Blood samples drawn before prostatectomy. No healthy controls tested.	Yes
Miyamoto 2018 [58]	34 (22 T1, 11 T2, 1 T3)	CTC-iChip + WTA + multiplex (8 genes) ddPCR	Mean CTC in healthy + 2SD of CTC score in healthy	ND	NA	Blood samples collected before surgery; 34 age-matched healthy donors included.	Yes
Murray 2018 [59]	241 (181 low risk + 60 intermed-iate risk)	Density gradient + PSA ICC	≥1 CTC/8 mL	37 low risk (20.4%) 26 intermedi-ate risk (43.3%)	ND	Blood samples collected 3 months after radiotherapy and stored 48 h at 4 °C.	Yes

^1^ ND = Not described. ^2^ NA = Not applicable. pT stage = Pathological tumor stage. CTC = Circulating tumor cells. PSA = Prostate-specific antigen. PSMA = Prostate-specific membrane antigen. PSCA = Prostate stem cell antigen. AAM = African Americans. CAM = Caucasian Americans. RT-PCR = Reverse transcription-polymerase chain reaction. EpCAM = Epithelial cell adhesion molecule. AOX = Antioxydant genes. FACS = Fluorescence-activated cell sorting. AR = Androgen receptor. AR-V7 = Androgen receptor splice variant seven. CK = Cytokeratins. Vim = Vimentin. N-Cad = N-Cadherin. WTA = Whole transcriptome amplification. ddPCR = droplet digital polymerase chain reaction. ICC = Imunocytochemistry. 2SD = Two times the standard deviation.

**Table 2:** The clinical value of Circulating Tumor Cells‘ (CTC) detection in patients with localized prostate cancer.

Study [ref]	Method	N° of Patients	N° of CTC+ Patients (%)	Mean Follow-Up Period	Type of Clinical Value (*P* Value)	Comments
Olsson 1996 [19]	PSA RT-PCR	100 (cT1–cT2c)	74 (74%)	13.6 months	Predictive of surgical failure (*P* < 0.0286)	Correlation of RT-PCR results before prostatectomy with disease recurrence after prostatectomy.
Ennis 1997 [20]	PSA RT-PCR	156 (cT1–cT2)	ND	ND	Prognostic (*P* < 0.0001)	Correlation of RT-PCR results with pathological stage and prediction of extra-capsular disease.
Mejean 2000 [24]	PSA RT-PCR	79 (cT1–cT2)	ND	26 months	Predictive (*P* < 0.04)	CTC detection associated with the development of metastases and risk of relapse after prostatectomy.
Slawin 2000	hK2 RT-PCR	154 (pT1–pT2)	ND	ND	Prognostic (*P* = 0.028)	Association with the risk of metastasis to pelvic lymph nodes.
Bianco 2002 [28]	PSA RT-PCR	96 (35 pT1, 61 pT2)	26 (27%) African Americans	33 months	Prognostic (*P* = 0.01)	Association with tumor stage and recurrence in African-Americans.
Yates 2012 [40]	PSA & PSMA RT-PCR	92 (61 pT1, 31 pT2)	63 (68.5%) PSA 68 (78.9%) PSMA	72 months	Predictive (*P* = 0.03)	Improved prediction of biochemical recurrence.
Puche-Sanz 2017 [54]	CK immune-magnetic	86 (pT1–pT2)	16 (18.6%)	ND	Theranostic & Diagnostic (*P* = 0.03)	Expression of AR in tumor tissue correlated significantly with presence of CTC in blood. Diagnosis of PCa by CTC has a 14.2% sensitivity and a 78.4% specificity.
Llanes 2000 [25]	PSA RT-PCR	25 (pT1–pT2b)	7 (28%)	ND	Not significant	The best predictors of extraprostatic disease were the biopsy Gleason score and the PSA level.
Shariat 2002 [27]	PSA RT-PCR HK2L	224 (pT1–pT2)	54 (24%)	52.9 months	Not significant	Preoperative blood RT-PCR-PSA not associated with characteristics or outcomes of prostate cancer HK2L correlation with risk of metastases.
Thomas 2002 [30]	PSA & PSMA RT-PCR	136 (pT1–pT2)	73 (54%)	59 months	Not significant	RT-PCR status did not predict pathologic stage or biochemical failure.
Helo 2009 [34]	PSA & PSMA RT-PCR	87 (cT1–cT2)	6 (7%)	28 months	Not significant	No association between KLK mRNA status and unfavorable localized disease features.

ND = Not described; cT1–cT2 = clinical stages T1–T2; pT1–pT2 = pathological stages T1–T2
